# ADAMTS3 activity is mandatory for embryonic lymphangiogenesis and regulates placental angiogenesis

**DOI:** 10.1007/s10456-015-9488-z

**Published:** 2015-10-07

**Authors:** Lauriane Janssen, Laura Dupont, Mourad Bekhouche, Agnès Noel, Cédric Leduc, Marianne Voz, Bernard Peers, Didier Cataldo, Suneel S. Apte, Johanne Dubail, Alain Colige

**Affiliations:** Laboratory of Connective Tissues Biology, Tour de Pathologie, GIGA-R, University of Liege, B23/3, 4000 Sart Tilman, Belgium; Laboratory of Tumor and Developmental Biology, GIGA-R, University of Liege, 4000 Sart Tilman, Belgium; Laboratory of Zebrafish Development and Disease Models, GIGA-R, University of Liege, 4000 Sart Tilman, Belgium; Department of Biomedical Engineering, Cleveland Clinic, Lerner Research Institute, Cleveland, OH 44195 USA

**Keywords:** ADAMTS, Lymphangiogenesis, Angiogenesis, Collagen, Placenta, Development

## Abstract

The only documented activity of a subclass of ADAMTS proteases comprising ADAMTS2, 3 and 14 is the cleavage of the aminopropeptide of fibrillar procollagens. A limited number of in vitro studies suggested that ADAMTS3 is mainly responsible for procollagen II processing in cartilage. Here, we created an ADAMTS3 knockout mouse (Adamts3^−/−^) model to determine in vivo the actual functions of ADAMTS3. Heterozygous Adamts3^+/−^ mice were viable and fertile, but their intercrosses demonstrated lethality of Adamts3^−/−^ embryos after 15 days of gestation. Procollagens I, II and III processing was unaffected in these embryos. However, a massive lymphedema caused by the lack of lymphatics development, an abnormal blood vessel structure in the placenta and a progressive liver destruction were observed. These phenotypes are most probably linked to dysregulation of the VEGF-C pathways. This study is the first demonstration that an aminoprocollagen peptidase is crucial for developmental processes independently of its primary role in collagen biology and has physiological functions potentially involved in several human diseases related to angiogenesis and lymphangiogenesis.

**Electronic supplementary material:**

The online version of this article (doi:10.1007/s10456-015-9488-z) contains supplementary material, which is available to authorized users.

## Introduction

ADAMTS (**AD**isintegrin **A**nd **M**etalloproteinase with **T**hrombo**S**pondin type 1 repeat) are secreted metalloproteases forming a family of 19 different members that share an identical N-terminal domain organization [1]. By contrast, they can be characterized or classified in subfamilies according to the presence of specific C-terminal ancillary domains or on the basis of their activities or substrates. Proteoglycanases (ADAMTS1, 4, 5, 8, 9, 15, 20) constitute a super-clade of ADAMTS able to cleave various proteoglycans although they possess a variable C-terminal domain organization [2]. ADAMTS13 is the only member reported to cleave the von Willebrand Factor [3–5]. ADAMTS2, 3 and 14 are highly related enzymes with identical domain organization, high sequence homology and shared ability to process the aminopropeptide of fibrillar collagens, categorizing them as “aminoprocollagen peptidases” [6].

Mutations in *ADAMTS2* are responsible for a human-inherited disease, the dermatosparactic type of Ehlers–Danlos syndrome [7, 8], characterized among other features by severe skin fragility and easy bruising. These clinical hallmarks are thought to result from the accumulation of type I and type III aminoprocollagen (collagen still retaining its aminopropeptide), which hinders collagen assembly into highly organized fibrils. Other significant findings relating to potential functions are the relative resistance of *Adamts2*^−*/*−^ mice to liver fibrosis [9], the infertility of *Adamts2*^−*/*−^ males [10], the expression of ADAMTS2 by activated macrophages [11], its anti-angiogenic properties [12] and its potential role in pediatric stroke [13].

In contrast to the extensive literature on ADAMTS2, ADAMTS3 remains much less characterized. It efficiently processes the aminopropeptide of procollagen II in vitro and is specifically expressed in cartilage during embryogenesis and adult life [14]. Previously, in situ hybridization (ISH) analysis revealed that *Adamts3* was also highly expressed in specific regions of the developing mouse brain and in connective tissues such as bone and tendon [14]. It was therefore assumed that ADAMTS3 would be the main aminoprocollagen peptidase during embryogenesis, which would explain the apparent normal development of these tissues in *Adamts2*^−*/*−^ embryos. In experimental models, ADAMTS3 was also recently shown to be able to activate VEGF-C by proteolytic cleavage [15] through a yet poorly characterized mechanism that probably involves also CCBE1, a secreted protein containing a collagenous domain. Indeed, CCBE1 is required for lymphangiogenesis by its ability to stimulate the activation of VEGF-C [16, 17] although having no proteolytic activity. It is not yet clear, however, whether this putative VEGF-C activating property of ADAMTS3 is physiologically relevant and restricted to ADAMTS3 since the highly similar ADAMTS2 and 14 could potentially share this function in vivo.

The aim of the present study was to elucidate the physiological roles of ADAMTS3 through *Adamts3* gene inactivation in mice. We show here that ADAMTS3 is dispensable for aminoprocollagen processing until mid-gestation. It is, however, critical for embryonic development since its deficiency prevents the formation of the lymphatic network, alters the function of placental blood vessels and drastically impairs liver development, which finally induces embryonic lethality. Our data demonstrate the central role of ADAMTS3 during two vital developmental processes and further show that the overlap of functions with ADAMTS2 is less important than previously expected.

## Results

### *Adamts3* inactivation leads to embryonic lethality with skin edema and impaired liver development, but procollagen processing is not altered

A mouse strain allowing the *Cre*-mediated conditional removal of the exon 8 to exon 10 *Adamts3* gene sequence was created. At the protein level, the skipping of these exons results in the absence of the catalytic metalloprotease domain, which prevents the possibility of any residual enzymatic activity, and introduces a frameshift by changing the *Adamts3* ORF (Fig. S1). It is worth mentioning also that this short genomic region does not contain any described miRNA or long noncoding RNAs. These mice were crossed with mice expressing *Cre* globally, including in the germ lines, in order to produce mice with a single functional *Adamts3* allele (*Adamts3*^+/−^). The shorter mRNA produced from the targeted allele was RT-PCR amplified and sequenced, confirming both the specific skipping of exons 8–10 and the reading frameshift of the mRNA. *Adamts3*^+*/*−^ mice were crossed, but no *Adamts3*^−*/*−^ neonates were observed in the litters, suggesting embryonic lethality. Genotyping of embryos at various development stages demonstrated that *Adamts3*^−*/*−^ embryos died around E15.0 and that the expected ratio between *Adamts3*^+*/*+^, *Adamts3*^+*/*−^ and *Adamts3*^−*/*−^ was Mendelian at earlier stages [25:56:19 % for E13–13.5 (*n* = 113) and 21:56:23 % for E14–14.5 (*n* = 281), 48:46:6 % for E15–15.5 (*n* = 31)].

**Fig. 1:**
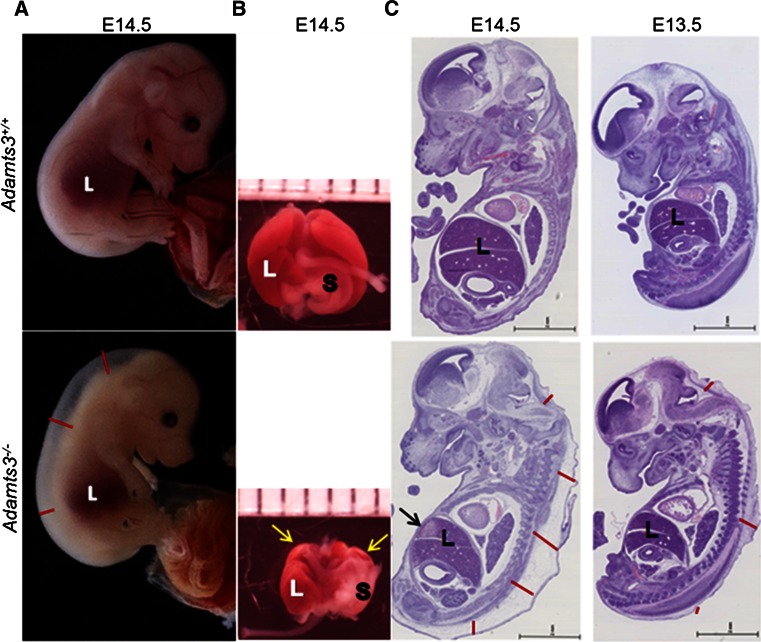
Characterization of *Adamts3* ^−*/*−^ embryos. **a** General morphology of *Adamts3* ^+*/*+^ and *Adamts3* ^−*/*−^ embryos at E14.5. Pale color, skin edema (*red lines*) and smaller liver (*L*) are evident in *Adamts3* ^−*/*−^ embryos. **b** General morphology of *Adamts3* ^+*/*+^ and *Adamts3* ^−*/*−^ livers from E14.5 embryos. Stomach (*S*) and liver (*L*) are indicated as well as degradation zones of the liver (*yellow arrows*). **c** Sagittal sections of whole embryos at E14.5 and E13.5 were stained with H&E, illustrating skin edema in E14.5 and E13.5 *Adamts3* ^−*/*−^ embryos (*red lines*). Reduction in liver (L) size and the presence of localized altered liver zones (*arrow*) are visible at E14.5

At E12.5, the gross morphological appearance of *Adamts3*^+*/*+^, *Adamts3*^+*/*−^*and Adamts3*^−*/*−^ embryos was similar, whereas *Adamts3*^−*/*−^ embryos could be identified at E13.5 by the presence of edema in the dorsal skin. Skin edema and a paler color were striking in E14.5 *Adamts3*^−*/*−^ embryos (Fig. 1a), as well as a reduction in liver size as observed after dissection (Fig. 1b). General examination of H&E-stained sections confirmed the progressive development of edema in *Adamts3*^−*/*−^ embryos from E13.5 to E14.5 and the reduction in liver size, while the other tissues and organs did not seem to be significantly affected (Fig. 1c).

Another relevant alteration was observed in blood-rich regions (heart, aorta, large blood vessels). At E14.5, the ratio of nucleated/nonnucleated red blood cells (RBC) was higher in *Adamts3*^−*/*−^ embryos and was confirmed on blood smears (Fig. S2). This observation further supported liver dysfunction, since the liver becomes the dominant hematopoietic organ at this developmental stage and produces nonnucleated RBC, while RBC produced earlier in the yolk sac are nucleated.

**Fig. 2:**
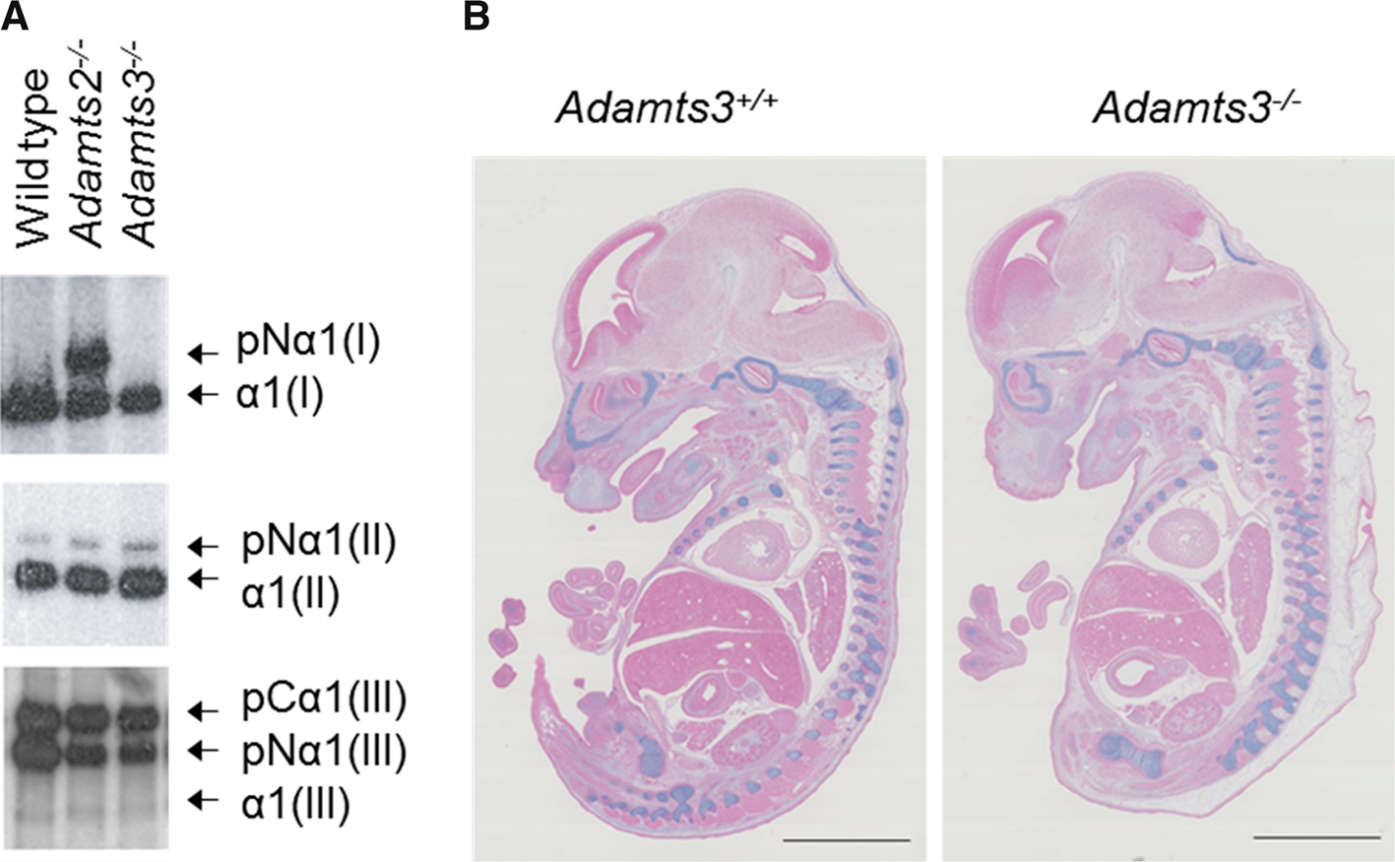
Fibrillar procollagen processing and cartilage visualization in *Adamts3* ^−/−^ embryos. **a** Connective tissue (body without head and viscera) of wild-type *Adamts2* ^−*/*−^and *Adamts3* ^−*/*−^embryos at E14.5 was homogenized and analyzed by Western blotting using antibodies specific for procollagens I, II or III. Modification of the levels of processed and unprocessed collagens was only seen for type I collagen in *Adamts2* ^−/−^ tissue. pCα: procollagen α chain; pNα: collagen α chain still retaining the amino-terminal extension; α: fully processed α chain. **b** Sections of *Adamts3* ^+*/*+^ and *Adamts3* ^−*/*−^ embryos were stained with *Alcian blue* to identify cartilage (*scale bar* 2 mm)

Since the best described substrates of ADAMTS3 are fibrillar procollagens [18], potential processing defects were therefore evaluated by Western blotting. *Adamts2*^−*/*−^ embryos were used for comparative purposes. As compared to WT, defects in the processing of the aminopropeptide of type I collagen was evident in *Adamts2*^−*/*−^, but not in *Adamts3*^−*/*−^ embryos (Fig. 2a), showing that ADAMTS2, and not ADAMTS3 as previously thought, is the main type I aminoprocollagen peptidase during embryogenesis. Regarding type II and type III collagens, the pattern was similar in the three genotypes, which suggested that ADAMTS2, ADAMTS3 and potentially ADAMTS14 would be capable of compensating their respective absence. The accumulation of type II collagen (not shown) and the formation of cartilage (Fig. 2b) were identical in *Adamts3*^+*/*+^ and *Adamts3*^−*/*−^ embryos.

### Abnormal lymphangiogenesis

**Fig. 3:**
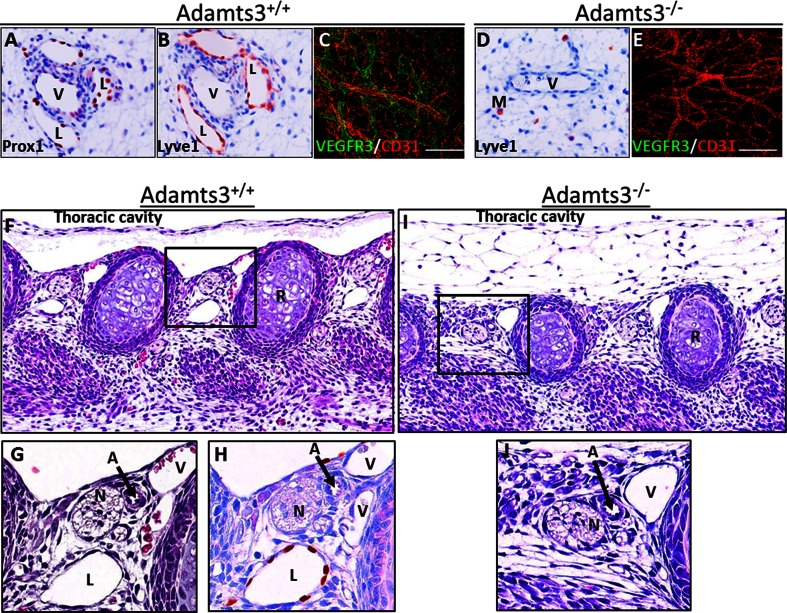
Normal angiogenesis but absence of lymphatics in *Adamts3* ^−*/*−^ embryos. Superficial sagittal sections in the skin of wild type (*Adamts3* ^+*/*+^) (**a**, **b**) and *Adamts3* ^−*/*−^ (**d**) E14.5 embryos were stained using antibodies specific to prox-1 (**a**) or Lyve-1 (**b**, **d**). While many lymphatics were identified in *Adamts3* ^+*/*+^ skin, they were never observed in *Adamts3* ^−*/*−^. *V* vein, *L* lymphatic, *M* macrophage. Full-thickness whole mounts of *Adamts3* ^+*/*+^ (**c**) and *Adamts3* ^−*/*−^ (**e**) dorsal skin were immunostained to identify blood vessels (CD31, in *red*) and lymphatics (VEGF-R3, in *green*). Lympatics were observed only in *Adamts3* ^+*/*+^ skin (see also Fig S3 for individual pictures and additional data). Sections at the border between the thoracic cavity and the rib cage (**f**–**j**) were also stained with H&E (**f**, **g**, **i**, **j**) or using an anti-prox-1 antibody (**h**). Enlarged views of the *boxed regions* in **f**, **i** are shown in **g**, **j**, respectively. Prox-1 staining (**h**) of the section following the section in **f** is also provided. Nerve (*N*), artery (*A*) and vein (*V*) are seen in *Adamts3* ^+*/*+^ and *Adamts3* ^−*/*−^. Lymphatics (*L*) are often seen in *Adamts3* ^+*/*+^ but never in *Adamts3* ^−*/*−^

Because skin edema was the first visible phenotype in *Adamts3*^−*/*−^ embryos, we first focused our attention on cutaneous lymphatics using superficial sagittal sections. Blood vessels and lymphatics were present in the skin of *Adamts3*^+*/*+^ embryos (Fig. 3a, b). A comparable number of veins and arteries were seen in the *Adamts3*^−*/*−^ skin samples, but not a single lymphatic was identified (Fig. 3d). Full-thickness whole mounts of the dorsal skin were also evaluated after immunofluorescence labeling of blood vessels (CD31, in red) and lymphatics (VEGF-R3, in green). The blood vessel network was similar in *Adamts3*^−*/*−^ and *Adamts3*^+*/*+^ embryos (Fig. 3c, e; see also Fig. S3 for additional pictures). In sharp contrast, while VEGF-R3-positive dermal lymphatic vessels were clearly visible in control embryos, none was observed in *Adamts3*^−*/*−^ skin, in agreement with immunohistochemical data.

Another highly specific and localized area where lymphatics can be clearly identified is the intercostal neurovascular bundle formed by a vein, an artery, a nerve and very often a lymphatic at that embryonic stage (Fig. 3f–h). Only veins, arteries and nerves were observed in the *Adamts3*^−*/*−^ embryos (Fig. 3i, j), further confirming the critical role of Adamts3 in lymphangiogenesis.

**Fig. 4:**
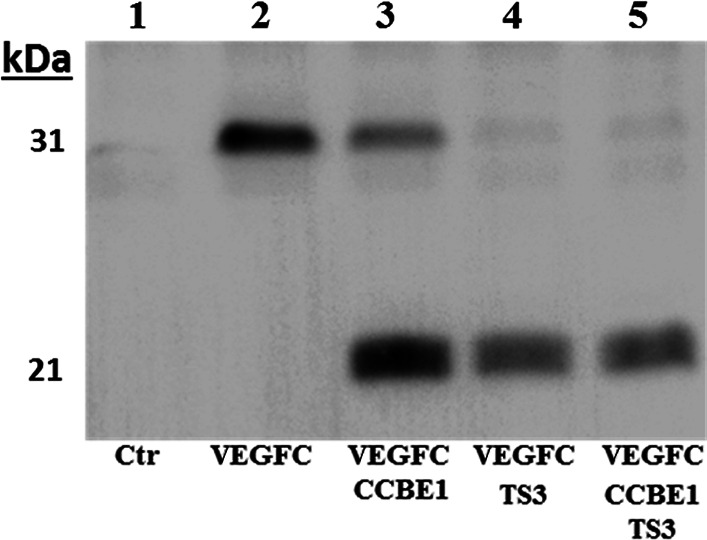
Activation of the intermediate form into the fully active form of VEGF-C by ADAMTS3. HEK293 cells were transfected with an empty vector (Ctr) or with an expression vector containing the complete coding sequence of VEGF-C (*lanes 2–5*). In some conditions, cells were simultaneously transfected with expression vectors for CCBE1 (*3*, *5*) and/or ADAMTS3 (*4*, *5*). Culture media were collected after 48 h and analyzed by WB. Conversion of the intermediate processed form of VEGF-C (31 kDa) into its mature form (21 kDa) is observed in the presence of CCBE1 alone and is almost complete in the presence of ADAMTS3

Since VEGF-C is a crucial growth factor for lymphatic formation and in line with recent data [15], we used a model of cotransfection to evaluate the ability of Adamts3 to activate “pro-VEGF-C.” As illustrated in Fig. 4, we found that Adamts3 promoted the activation of VEGF-C, even in the absence of recombinant CCBE1. Using the same model, Adamts2 and Adamts14 were not able to activate VEGF-C in the presence or absence of CCBE1 (not shown). VEGF-C is similarly expressed in *Adamts3*^+/+^ and *Adamts3*^−/−^ embryos, as judged from qRT-PCR (not shown). Crude tissue extracts were also analyzed by Western blotting, an approach that turned out to be not sensitive enough to allow the detection of VEGF-C, either as pro-form or as the fully activated form (not shown). An enrichment protocol using soluble VEGF-R3 as bait was therefore designed to determine the activation status of VEGF-C in these embryos. Despite the fact that control samples (tissue extract supplemented with 10 ng of recombinant mouse VEGF-C) were positive, again no VEGF-C signal could be observed in experimental samples. Based on the reported active concentration range of VEGF-C in blood and in the extracellular compartment (0.1–2.5 ng/ml), the total amount of VEGF-C in the entire embryo (embryo weight < 300 mg) is most probably largely under the detection limit by Western blotting.

### Hepatic architecture deteriorates after E13.5

**Fig. 5:**
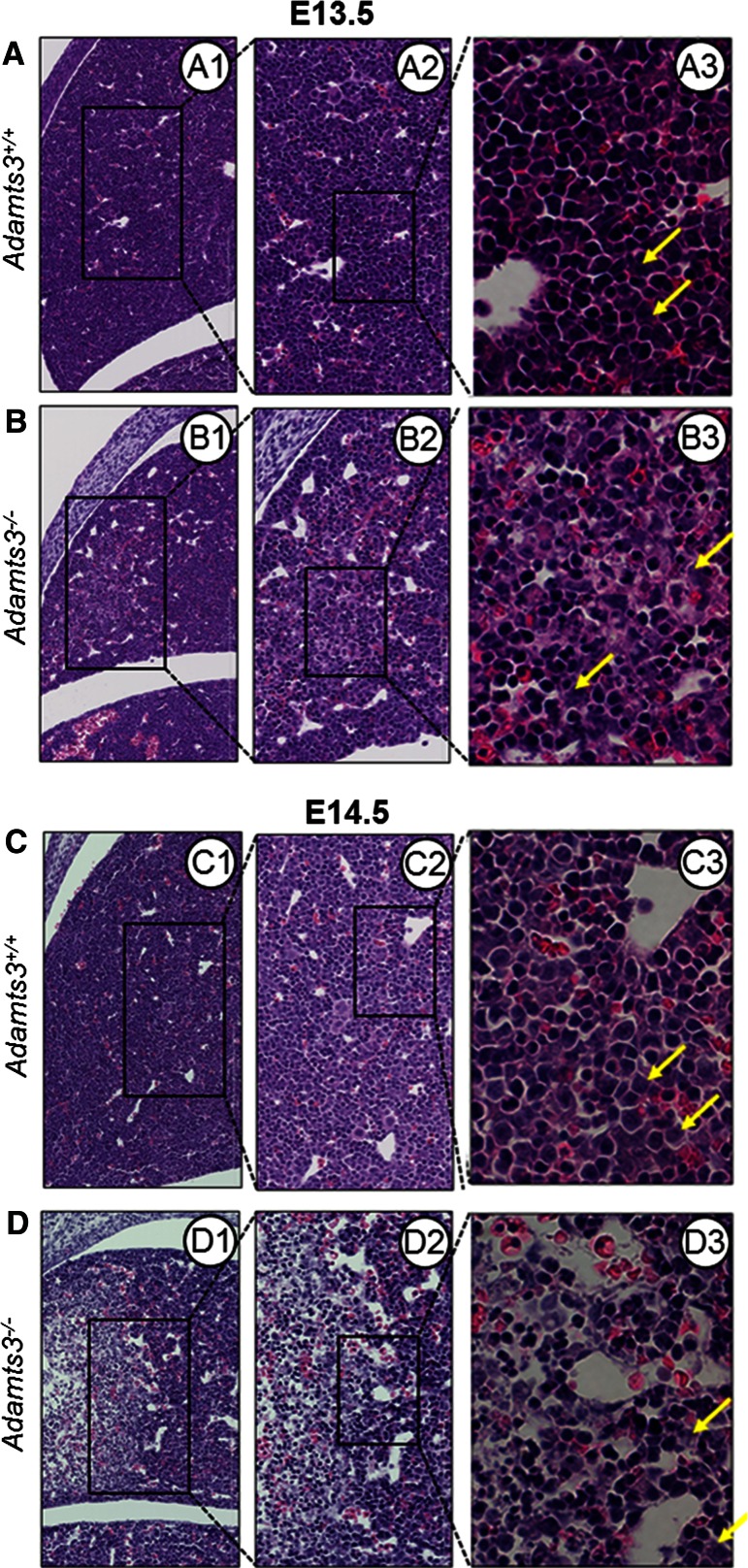
Evaluation of *Adamts3* ^−*/*−^ liver defects. Photomicrographs of HE-stained sections of whole embryos (*Adamts3* ^+*/*+^ **a**, **c**; *Adamts3* ^−*/*−^ **b**, **d**) at E13.5 (**a**, **b**) and E14.5 (**c**, **d**) were taken in identical regions at increasing magnifications. Hepatoblasts (*yellow arrows*) appear as *light purple* cells containing a large, weakly stained nucleus. A large proportion of blood cells (red blood cells and small rounded cells with a strongly stained nucleus) are also visible throughout the parenchyma. At E13.5, some hepatocytes in focal areas of *Adamts3* ^−*/*−^ livers are swollen (**B3**), while they seem normal at short distance. At E14.5, no hepatoblasts are still present in the center of the lesions and only a reduced number are visible at the periphery, whereas many blood cells are still present

Before E13.0, liver histology was comparable in the *Adamts3*^+*/*+^ and *Adamts3*^−*/*−^ embryos. By E13.5, a few abnormal swollen cells systematically appeared in highly localized ventral areas of *Adamts3*^−*/*−^ livers (compare Fig. 5a, b). At E14.5, stronger alterations were apparent (Fig. 5c, d), which suggested a progressive spreading from the initial lesion.

Sections of whole embryo were further characterized by immunohistochemistry and immunofluorescence, with a more specific focus on the liver. In *Adamts3*^+*/*+^ embryos, very few hepatic cells were apoptotic (cleaved caspase 3-positive staining) (Fig S4A), while *Adamts3*^−*/*−^ livers were characterized by a strong positivity in the ventral portion of the lobes, which progressively enlarged from E13.5 to E14.5. Diffuse labeling through the entire liver was never observed. KI67 staining of proliferating cells was similar in all the tissues of the three genotypes, except in the affected zones of the liver (Fig. S4B). Of special interest, blood vessels appeared enlarged in the ventral part of the liver lobes (Fig. S4C), even before the first clear signs of apoptosis.

In light of the observed skin and liver damages, RNA ISH using a new and sensitive method was used to re-evaluate the distribution of *Adamts3* mRNA in embryos at E13.5 and E14.5. As previously identified, strong expression was seen in specific regions of the central nervous system, craniofacial region and limbs (Fig. S5A) and in cartilage of the ribs (Fig. S5B). Expression of *Adamts3* was also detected at E13.5, although at a lower level, in dermal mesenchyme of skin (Fig. S5C) but only in scattered liver cells (Fig. S5D). RT-PCR amplification was also performed as an additional control to confirm *Adamts3* expression in the liver and the “body.” Products of expected size were obtained for both tissues (Fig. S5E), with a relative expression correlating with the ISH data.

### Transcriptome analyses

In an attempt to identify the pathway(s) affected in *Adamts3*^−*/*−^ liver, microarray analyses were performed using hepatic tissues of E13.5 to E14.5 embryos in order to determine the early and late events affected by *Adamts3* deficiency (ArrayExpress: E-MTAB-2614). The reliability of the transcriptomic data was confirmed by RT-PCR (Fig. S6).

At E13.5, only *Esm1* and *Xlr4a* were significantly upregulated (>2) in Adamts3^−/−^, while 8 of the 11 repressed genes (*Ear2, 3, 4, 6, 10, 12; Prg2/*MBP-1; *Prg3/*MBP-2) were related to the eosinophil lineage (Table S1). At E14.5, the number of upregulated or downregulated genes dramatically increased in agreement with the deep deteriorations occurring in the liver just before embryonic death. As these massive modifications hampered the identification of the primary causes of the phenotype, we focused our attention on genes significantly expressed (>200 A.U.) in at least two samples and characterized by a consistently progressive increased or decreased expression correlating with the severity of the alterations observed in *Adamts3*^−*/*−^ livers. This filtering provided a limited number of genes that were all characterized by a progressively increased expression (Table 1; S2). A majority of these genes were related to one or two of the following categories: “connective tissues,” “angiogenesis,” “inflammation,” “signaling pathways” and “glucose metabolism.” The earliest and most upregulated gene was ESM1, a marker of activated endothelial cells. Flt1 (VEGF-R1) and VEGF-A, two other factors regulating angiogenesis, were also upregulated. The most abundant group (glucose metabolism) comprised factors directly involved in glycolysis, glucose uptake (Glut3/Slc2a3) and intracellular signaling pathways regulating glucose metabolism (Trib3 and Egln3/Phd3, the second and third most upregulated genes after ESM1). Although the fold change remained moderate for some enzymes and factors, the overrepresentation of this well-defined category (20 out of 89 transcripts) was striking.

**Table 1:** Main functional categories of genes identified by microarray analysis as characterized by a progressive increased or decreased expression correlating with the severity of liver alterations

Angiogenesis	*Egln3/PHD3, Eif4ebp1, Esm1, Flt1, Pecam/CD31, Vegf-a*
Connective tissues	*Adamts2, Col1a1, Col5a1, Col6a1, Col6a3, Ctgf, Lamc1, Lgals3, P4ha2*
Glucose metabolism/glycolysis	*Adh1, Aldoa, Gtl2, Egln3/PHD3, Egr1, Eno, Fam132b, Gapdh, Gpi1, Grhpr, Ldha, PDK1, PFKl, PFKp, PKM2, Pgam1, Pgk1, Slc2a3/Glut3, Stat3, Tpi, Trib3*
Inflammation	*Ctgf, Ctsg, Cxcl1, Cxcl10, F13a1, Mpo, Nfkbiz, S100a9, Stat3, Vav1, Lcn2*
Signaling pathways	*Cebpb, Ctgf, Cxcl1, Cxcl10, Dusp6, Egln3/PHD3, Egr1, Ets2, Gata6, Junb, Mapkapk3, Mt2, Stat3, Trib3, Vav1*

See Table S2 for the complete list and values

Although many genes and categories identified by transcriptomic analyses were known targets of the TGFβ pathways, no significant modification of the mRNA level of TGFβ was identified. A significant increase in TGFβ1 was, however, observed by Western blotting in crude extracts of *Adamts3*^−*/*−^ livers at E14.5 (Fig. S7). The potential implication of TGFβ1 in the phenotype was therefore investigated by IHC staining of phospho-Smad2 and phospho-Smad3 in liver sections, but no clear difference between *Adamts3*^+*/*+^ and *Adamts3*^−*/*−^ samples could be identified, which suggested that TGFβ1 accumulation was not the main cause of liver apoptosis.

### Blood vessels are altered in the placenta of homozygous mutants

**Fig. 6:**
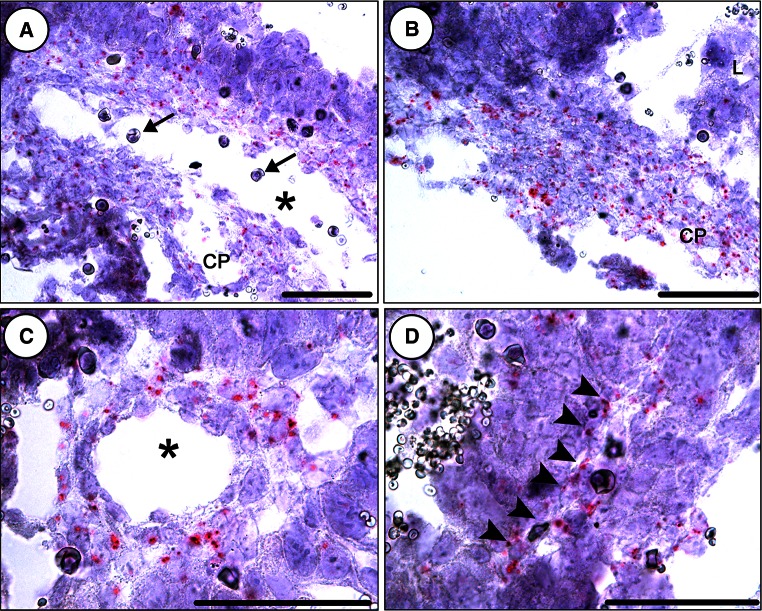
Evaluation of Adamts3 expression in mouse placenta. Sections of E12.5 placenta were hybridized with *Adamts3* probes (see Fig. S8 for additional details) and counterstained with Gill’s hematoxylin. Nuclei are stained in *blue,* and the expression of *Adamts3* is revealed by the presence of *red dots*. The strongest *Adamts3* expression was observed in the chorionic plate (**a**, **b)**, the structure where large embryonic vessels (*asterisk*) enter the placenta. Nucleated erythroblasts are marked by *arrows*. Positivity is also observed in the labyrinthine layer, mainly around large vessels (**c**) and along structures looking like small blood vessels (*arrowheads* in **d**). Only a limited number of trophoblastic cells, identified by their very large nucleus, are faintly positive. *CP* chorionic plate, *L* labyrinthine layer. *Scale bars* 50 µm

Since many genes upregulated in *Adamts3*^−*/*−^ livers were linked to hypoxia and glycolysis, we made the hypothesis that it might be related to placenta alterations that would result in insufficient oxygen and nutrient supply to the embryo. RT-PCR amplifications showed that *Adamts3* is expressed in placenta. RNA ISH was next used to identify cells expressing Adamts3. Only a limited number of trophoblastic cells were faintly positive, and the strongest staining was observed in the chorionic plate (Fig. 6a, b; see also Fig S8). Positivity was also observed in the labyrinthine layer but mainly around large vessels and along structures looking like small blood vessels (Fig. 6c, d), which suggested that mesodermal/mesenchymal cells are the main producers of Adamts3.

**Fig. 7:**
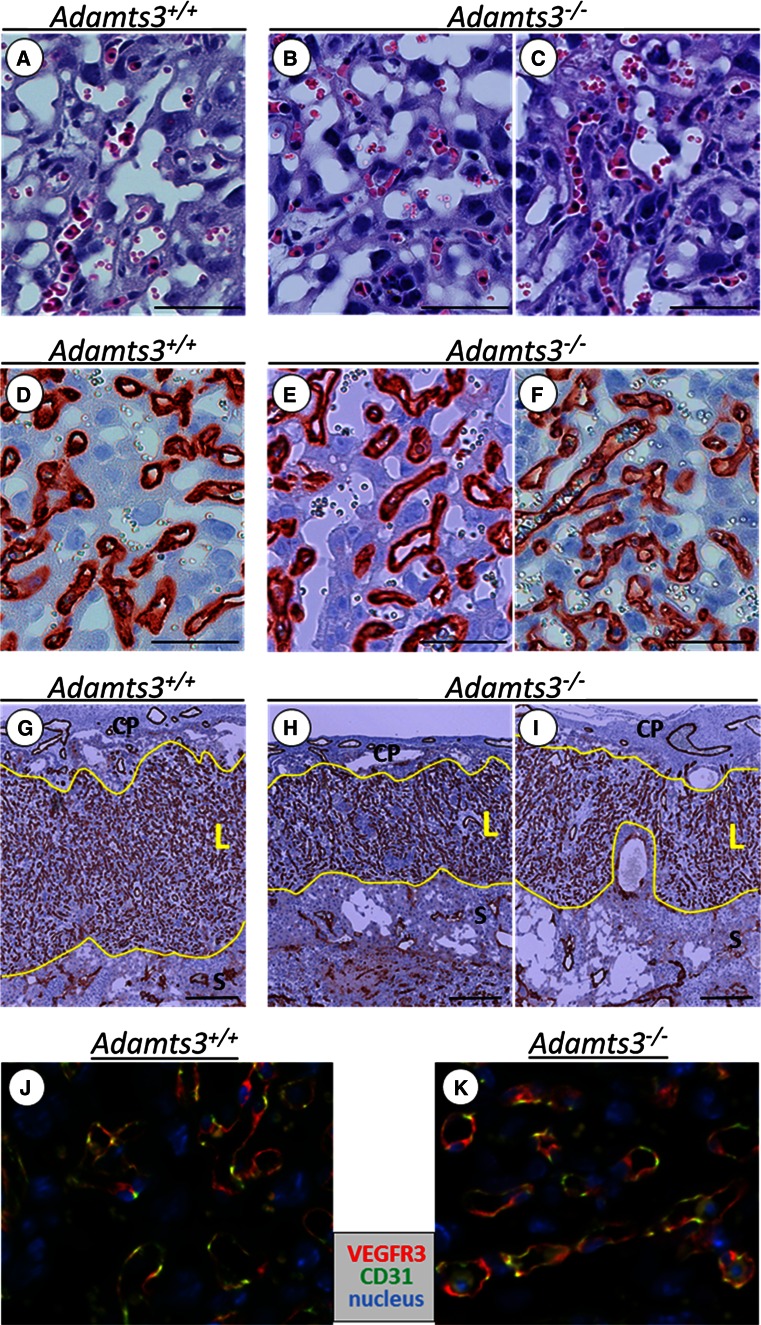
Immunohistologic analysis of placental blood vessels. Transverse sections were performed in E14.5 placenta (*Adamts3* ^+*/*+^ in **a**, **d**, **g**, **j**; *Adamts3* ^−*/*−^ in **b**, **c**, **e**, **f**, **h**, **i**, **k**). All the pictures were taken in the labyrinthine layer where the blood vessels containing embryonic blood are present. After H&E staining (**a**–**c**), the structure of the *Adamts3* ^−*/*−^ placenta seemed grossly normal (compare **b** to **a**), except for the accumulation of nucleated erythroblasts in some vessels (**c**). CD31 staining (**d**–**f**) showed a normal pattern of blood vessel distribution in *Adamts3* ^−*/*−^ placenta but suggested some reduction in vessel diameter (**d**–**f**). Transverse sections were also performed in the middle of other placenta (*Adamts3* ^+/+^ in **g**; two different *Adamts3* ^−/−^ placenta in **h** and **i**; see also Fig. S10 for additional data). At low magnification, the labyrinthine layer (*L* delineated by *yellow lines*) is clearly identified by its high blood vessel density. Its surface is significantly reduced in Adamts3^−/−^ placenta. Immunofluorescence staining (**j**, **k**) showed that blood vessels are formed by cells with different levels of expression of CD31 (*green*) and VEGF-R3 (*red*). No difference was found between *Adamts3* ^+*/*+^ and *Adamts3* ^−*/*−^ samples. *Bars* 50 µm in **a**–**f** and 300 µm in **g**–**i**. *CP* chorionic plate, *S* spongiotrophoblast layer

HE-stained sections looked quite similar in *Adamts3*^+*/*+^ and Adamts3^−/−^ placentas (Fig. 7a, b), except for the presence, in some regions of *Adamts3*^−*/*−^ placenta, of an increased number of fetal blood vessels that were full of nucleated red blood cells and that seemed too narrow to allow free flow (Fig. 7c). This observation was confirmed by CD31 (Fig. 7d–f) and basement membrane (collagen type IV, Fig. S9 A–C) staining. The mean diameter of the blood vessels was also determined with the NDP software (Hamamatsu) on four pairs of placenta from four different litters (Table S3, see legend for quantification details). A slight but significant reduction (14 %, *p* < 0.01) in the vessel diameter was found in *Adamts3*^−*/*−^ (8.6 ± 0.7 µ, *n* = 799) as compared to *Adamts3*^+*/*+^ (10.0 ± 0.6 µ, *n* = 1078). It is worth noting that in 2 litters the number of measurable vessels with a patent circular lumen was reduced in the *Adamts3*^−*/*−^ placenta, which suggested that some vessels had already collapsed and were not taken into account. Cross sections were further realized in the center of placenta from three different litters (3 *Adamts3*^−*/*−^ and 5 *Adamts3*^+*/*+^) in order to evaluate the respective thickness of the labyrinthine and the spongiotrophoblast layers, which can be easily discriminated by CD31 staining of the vessels containing the embryonic blood (Fig. 7g–i; Fig. S10). The mean total surface of the two layers was identical in the two genotypes (7.3 ± 1.5 mm^2^ in *Adamts3*^−*/*−^ vs 7.1 ± 0.9 mm^2^ in *Adamts3*^+*/*+^). However, the specific surface occupied by the labyrinthine layer was significantly reduced in *Adamts3*^−*/*−^ placenta (48.8 ± 3.3 vs 62.3 ± 2.0 % in *Adamts3*^+*/*+^).

In order to investigate why the blood vessels are not damaged in the Adamts3^−/−^ embryos while angiogenesis is modified in their placenta, we evaluated the potential expression of VEGF-R3 in placenta blood vessels. As we previously showed (Fig. 2c), VEGF-R3 staining was absent from blood vessels in E14.5 embryos. By contrast, endothelial cells in the placenta were positive for VEGF-R3, irrespective of the genotype (Fig. S9 D–F), which would explain why *Adamts3*^−*/*−^ placental vessels are affected, while vessels in the embryo are not. Faint staining of trophoblast clusters in the labyrinthine layer was also observed. Immunofluorescence studies further showed that almost all the blood vessels in the labyrinthine layer were formed by endothelial cells expressing VEGF-R3 and/or CD31, in both *Adamts3*^+*/*+^ and *Adamts3*^−*/*−^ placentas (Fig. 7j, k).

## Discussion

It was initially considered that the function of ADAMTS3 was exclusively or primarily related to procollagen processing [14, 18]. We developed an *Adamts3* knockout mouse model to evaluate this hypothesis and to identify potential additional functions in vivo. Mice with a single inactive allele (*Adamts3*^+*/*−^) were fertile, but their mating did not produce *Adamts3*^−*/*−^ pups because of lethality occurring around the E15.0 period. The processing of the main fibrillar collagens (types I, II and III) and their tissue localization were similar in *Adamts3*^−*/*−^ and *Adamts3*^+*/*+^ embryos, demonstrating that the strong expression of ADAMTS3 during embryonic development and the observed phenotypes in *Adamts3*^−*/*−^ embryos were not discernibly related to collagen biology. Two main phenotypes were observed in *Adamts3*^−*/*−^ embryos. Cutaneous lymphedema was striking and caused by the absence of lymphatics normally developing in the dorsal skin around E13.0–E13.5. Liver degeneration was also remarkable. It started around E13.5 in strictly localized ventral regions of *Adamts3*^−*/*−^ livers and then rapidly spread from this initial spot. The vast majority of the other mouse models of embryonic lymphedema do not display liver pathology [19, 20], and conversely, mouse strains characterized by severe hepatic alterations during embryogenesis do not display concomitant edema [21–24]. However, there is one gene for which deficiency leads to a phenotype displaying many similarities with the *Adamts3*^−*/*−^ phenotype. CCBE1 (Collagen- and Calcium-Binding EGF domain-containing protein 1) is mutated in a cohort of patients with Hennekam syndrome who present abnormal lymphangiogenesis [25]. In the mouse, Ccbe1 deficiency leads to embryonic lethality, edema and liver hypoplasia [17, 26]. As in *Adamts3*^−*/*−^ embryos, lymphedema is massive and similarly characterized by the absence of lymphatics normally forming around E13–E13.5. CCBE1 is required for lymphangiogenesis because it increases the processing of pro-VEGF-C into its fully active form able to stimulate VEGF-R3 phosphorylation and downstream signaling [16]. Very recently, it was shown in experimental models that CCBE1, although lacking intrinsic enzymatic activity, facilitates pro-VEGF-C activation by ADAMTS3, but not by its closest relatives ADAMTS2 and ADAMTS14 [15]. It was not clear, however, whether ADAMTS3 is required in physiological conditions or whether other proteases could be involved in vivo. Our data clearly demonstrate that ADAMTS3 is indispensable for embryonic lymphangiogenesis, which is most likely related to its capacity to activate VEGF-C.

While the lymphatic phenotype is identical in *Adamts3*^−*/*−^ and *Ccbe1*^−*/*−^ embryos, some similarities and differences were evidenced for liver. Hepatic alterations appear in both genotypes around E13.0–E13.5 [26]. This is accompanied by a marked increase of the nucleated/enucleated erythrocytes ratio in the blood stream, which is likely caused by hepatic disorders since the liver becomes the most important organ for erythropoiesis at mid-gestation. In *Ccbe1*^−*/*−^ embryos, however, the pattern of apoptosis was not reported to be as localized as seen in *Adamts3*^−*/*−^ and the reduction in the liver size was attributed primarily to altered erythropoiesis [26], whereas hepatoblasts are amongst the first dying cells in *Adamts3*^−*/*−^ embryos. Another specific observation in *Adamts3*^−*/*−^ livers was the enlargement of blood vessels surrounding the apoptotic area, even at E13.5 when the hepatic lesion is still highly localized to a limited number of cells.

Transcriptome analyses were performed on strongly (E14.5), mildly (E14.0) or almost unaffected (E13.5) livers in order to identify the molecular mechanism leading to apoptosis in the hepatic tissue. We focused our attention on genes characterized by a progressive increased or decreased expression correlating with the severity of the liver alterations observed in *Adamts3*^−*/*−^ embryos. Categories related to “connective tissues,” “angiogenesis,” “glucose metabolism” and “inflammation” were identified. Several secreted or intracellular factors related to cell regulation (“signaling pathway”) were also upregulated, some of them (Trib3, CTGF/CCN2, Egr1, Stat3…) having been linked to inflammation, tissue repair, liver fibrosis and TGFβ signaling [27, 28]. Since *Tgfbr3*^−*/*−^ embryos are not viable and display hepatic defects at mid- to late gestation [22], the implication of the TGFβ pathway was investigated. As compared to their control littermate, accumulation of TGFβ1 was specifically found in the liver of *Adamts3*^−*/*−^ embryos. However, this was not accompanied by increased phosphorylation of Smad2 or Smad3, which suggested that abnormal regulation of the TGFβ pathway was not the main cause of the hepatic phenotype. We next focused our attention on the categories of genes involved in “Angiogenesis” and in “Glucose metabolism/glycolysis” pathways. A common characteristic of many of these genes is that they are directly (*ESM1*, *VEGF*-*R1*, *VEGF-A*, *PHD3*) or indirectly (glycolysis enzymes, transcription factors regulating metabolic activity, such as *Egr1* and *Trib3*) responsive to hypoxia or deprivation of nutrients. Since blood vessels looked normal in embryos with no obvious sign of leakage or hemorrhage, we reasoned that the primary defect might reside in the placenta, a tissue allowing O_2_ and nutrient exchanges between the maternal and the fetal blood. In line with this hypothesis, it was previously shown that HGF deficiency causes placental defects that secondarily lead to a specific pattern of liver apoptosis always initially localized in the ventral part of the lobes, most probably because this area is at the extremity of the liver blood vessel network and therefore the most sensitive to oxygen supply and hypoxia [29, 30]. Tissue staining showed that the labyrinthine layer containing the embryonic blood is reduced in *Adamts3*^−*/*−^ placenta and that the CD31-positive vessels have a reduced diameter as compared to the *Adamts3*^+*/*+^ controls (8.6 vs 10.0 µm). Although this reduction is modest, it can be hypothesized to be functionally significant because nucleated erythroblasts that predominate in *Adamts3*^−*/*−^ embryo have an 8- to 9-µm mean diameter. In these conditions, even a small reduction in size is expected to restrict the bloodstream and, combined with a thinner labyrinthine layer, to result in a reduced oxygenation of the embryo. Since the embryonic liver is highly metabolic and at the limit of hypoxia during normal development, especially at the extremity of its vascular bed in the ventral parts of the lobes, it was not surprising to observe hypoxic markers only in the liver. This hypothesis is further strengthened by our observations, showing that blood vessels are enlarged in the ventral part of the lobes before the first clear signs of hepatic destruction. In the mutant embryos, reduced blood volume due to lymphedema and reduced vessel diameter in the placenta would act synergistically to lower nutrients and oxygen delivery to the embryo, which would primarily affect the liver and would further compromise oxygenation by reducing the formation of mature erythroblasts in the liver. This combination of events would explain why a Mendelian proportion of *Adamts3*^−*/*−^ embryos was observed before E14.5, while most of them were dead after E15.0.

The expression of VEGF-R3 in endothelial cells forming placental capillaries further illustrates that the ADAMTS3/VEGF-C/VEGF-R3 pathway is critical for placenta blood vessel at this embryonic stage. In this context, it would be interesting to characterize the blood vessel structure in the *Ccbe1*^−*/*−^ placenta. It is worth mentioning also that *Ccbe1* deficiency is less severe (lethality at E16.5) [17] than inactivation of *Adamts3* (lethality at E15.0). It will have to be determined whether this is related to the fact that pro-VEGF-C processing by ADAMTS3 occurs in the absence of Ccbe1, although with a reduced efficacy, while the reverse is not true. Alternatively, it could be hypothesized that the more severe phenotype in *Adamts3*^−*/*−^ results from other functions (procollagen processing, TGFβ pathway) that would per se only modestly affect embryos but that would synergize to aggravate the VEGF-C-related phenotype.

This study is the first demonstration that an aminoprocollagen peptidase is involved in collagen-independent processes in physiological conditions. We demonstrated that ADAMTS3 is required for embryonic lymphangiogenesis and placenta angiogenesis. These data identify ADAMTS3 as a candidate gene for genetic diseases characterized by lymphedema and/or placenta defects. Since total absence of ADAMTS3 in humans would likely lead to miscarriage, only hypomorphic mutations may be observed in genetic lymphedema. It would be most interesting also to evaluate whether ADAMTS3 is also involved in lymphatics homeostasis during the adulthood and in the abnormal lymphangiogenesis observed in several pathological conditions, including cancer and metastasis dissemination. The expression of ADAMTS3 in the brain and the implication of the VEGF-C/VEGF-R3 pathway in the activation of neural stem cells [31] suggest also a potential role for ADAMTS3 in neurogenesis.

## Materials and methods

### Transgenic mice

Mice were maintained under standard laboratory conditions, with 12-h light/12-h dark cycles and free access to food and water. All procedures were performed in accordance with the guidelines for animal care of the University of Liège, the Federation of European Laboratory Animal Science Associations and the American National Institutes of Health.

Genotyping of tail DNA was performed with KAPA Express Extract Kits (KAPA Biosystems, USA) using the following primer pairs: P1–P2 for wild-type allele; P3–P2 for the targeted allele; P4–P2 for the deletion of the Neo cassette inserted into intron 10; P5–P6 for *Flp* recombinase (Table S4).

The gestational age of embryos was determined from a vaginal plug, corresponding to 0.5 days postcoitum (E0.5). Pregnant females were killed by cervical dislocation and decapitation according to institutional guidelines. The uterus was removed and washed with physiological saline. Embryos were dissected free of decidual tissue and washed to remove contaminating maternal blood. After removal of the placenta, embryos were rinsed in saline, exsanguinated by separation of the vitelline and umbilical vessels and allowing blood to drain. Blood was smeared on glass slide and air-dried for May–Grunwald–Giemsa staining.

### Immunohistological analyses

Embryos or dissected organs were fixed in a 4 % paraformaldehyde (PFA) solution. Paraffin-embedded tissue sections (5 μm) were deparaffined and rehydrated. Tissue sections were stained with hematoxylin/eosin for general histology or with alcian blue (GURR, UK) for cartilage.

Fibrillar collagen was stained with antibodies against Col I [α1(I), 234167, Calbiochem, Germany], Col II [α1(II), SAB4500366, Sigma, USA] and Col III [α1(III), in-house developed guinea pig serum]. Blood and lymphatic vessels were identified using, respectively, anti-CD31 (Dia310, Dianova, Germany) or antibodies against LYVE1 (07-538, Upstate, USA), Prox1 (AF2777, R&D Systems) and VEGF-R3 (AF743, R&D Systems). The other antibodies were from AbD Serotec (F4/80, MCA497BB, AbD Serotec, UK), Cell Signaling Technology (USA) (Cleaved Caspase-3, 9664), Dako (Denmark) (Ki67, M7246) or Abcam (UK) (TGFβ1, ab66043).

### In situ hybridization

Placenta and mouse embryos were fixed overnight in 4 % paraformaldehyde/PBS, embedded in paraffin and sectioned (6 μm). In situ hybridization was carried out using the RNAscope technique and custom-designed in situ hybridization probes (RNAscope 2.0; Advanced Cell Diagnostics, Hayward, CA) according to the manufacturer’s instructions. Target probes were detected using an alkaline-phosphatase-conjugated labeled probe with Fast Red as substrate and Gill’s Hematoxylin as a counterstain. As a negative control, some sections were hybridized with target probe against *DapB*, a bacterial gene encoding dihydrodipicolinate reductase. A target probe directed against ubiquitously expressed *Polr2a* served as a positive control. The slides were then counterstained according to the manufacturer’s protocol with Gill’s hematoxylin and treated with ammonium water before mounting. In these conditions, nuclei are stained in blue and the expression of Adamts3 is revealed by the presence of red dots.

### Staining of blood and lymphatic vessels in whole mount skin

Whole embryos were fixed for 1 h in 70 % ethanol. The dorsal skin was then dissected and postfixed overnight in 4 % PFA. The samples were washed twice in PBS containing 0.2 % Triton X-100 (PBT) 30 min at 4 °C and blocked for 1 h in PBT containing 1 % BSA at RT. To perform double immunostaining, primary antibody against CD31 (at 1/150) and VEGF-R3 (at 1/50) (AF743, R&D Systems, USA) was diluted in PBT/BSA and incubated overnight at 4 °C. After three washings of 30 min in PBT at 4 °C and two at RT, the skin samples were incubated overnight at 4 °C with Alexa 488 anti-goat (1/1000 in PBT/BSA) and Alexa 546 anti-rat (1/1000) (Invitrogen, USA). After washing (as above), samples were mounted on glass plates with Aquapolymount (PolySciences Inc., USA) and observed with a Leika SP5 confocal microscope, adapted from [32].

### Western blotting

Liver and “body” (connective tissue remaining after careful removal of the head and viscera) were dissected, ground at liquid nitrogen temperature, homogenized in Laemmli denaturation buffer (with or without 100 mM DTT) and denatured (95 °C, 5 min). After electrophoresis (7.5–12.5 % SDS-PAGE), proteins were transferred onto a polyvinylidene difluoride membrane (NEN Life Sciences Products, USA). Membranes were blocked with 3 % BSA in PBS-Tween and were probed with a rabbit antiserum directed against type I, type II or type III collagens, or against TGFβ1. The secondary peroxidase-conjugated antibodies were swine anti-rabbit or rabbit anti-guinea pig immunoglobulins (1/2000, Dako). Peroxidase was revealed with an enhanced chemiluminescence assay (Pierce, USA) and X-ray film (Kodak, USA) exposure.

### Microarray analysis

RNA was extracted with NucleoSpin RNA/protein kit (Machery-Nagel, Germany) according to the manufacturer’s instructions. The RNA quality was evaluated with Eukaryote Total RNA Nano Assay of the Agilent 2100 Bioanalyzer (USA). Transcriptome analyses were performed on Illumina Mouse WG-6 v2.0 chips on total RNA from the liver of *Adamts3*^+*/*+^ and *Adamts3*^−*/*−^ littermates between E13.5 and E14.5. The raw data were analyzed using the GeneChip Operating software. Additional analyses were also performed with Ingenuity Pathway (Qiagen, USA) or DAVID software [33].

## Electronic supplementary material

Supplementary material 1 (DOCX 9544 kb)

Supplementary material 2 (DOCX 58 kb)
