# Supplementary material for: ADAMTS3 activity is mandatory for embryonic lymphangiogenesis and regulates placental angiogenesis
Source: Angiogenesis. 2015 Oct 7;19:53–65. doi: 10.1007/s10456-015-9488-z (PMC4700087; doi:10.1007/s10456-015-9488-z)
Supplement: Supplementary file 1 — Supplementary material 1 (DOCX 9544 kb) [file 10456_2015_9488_MOESM1_ESM.docx]

##
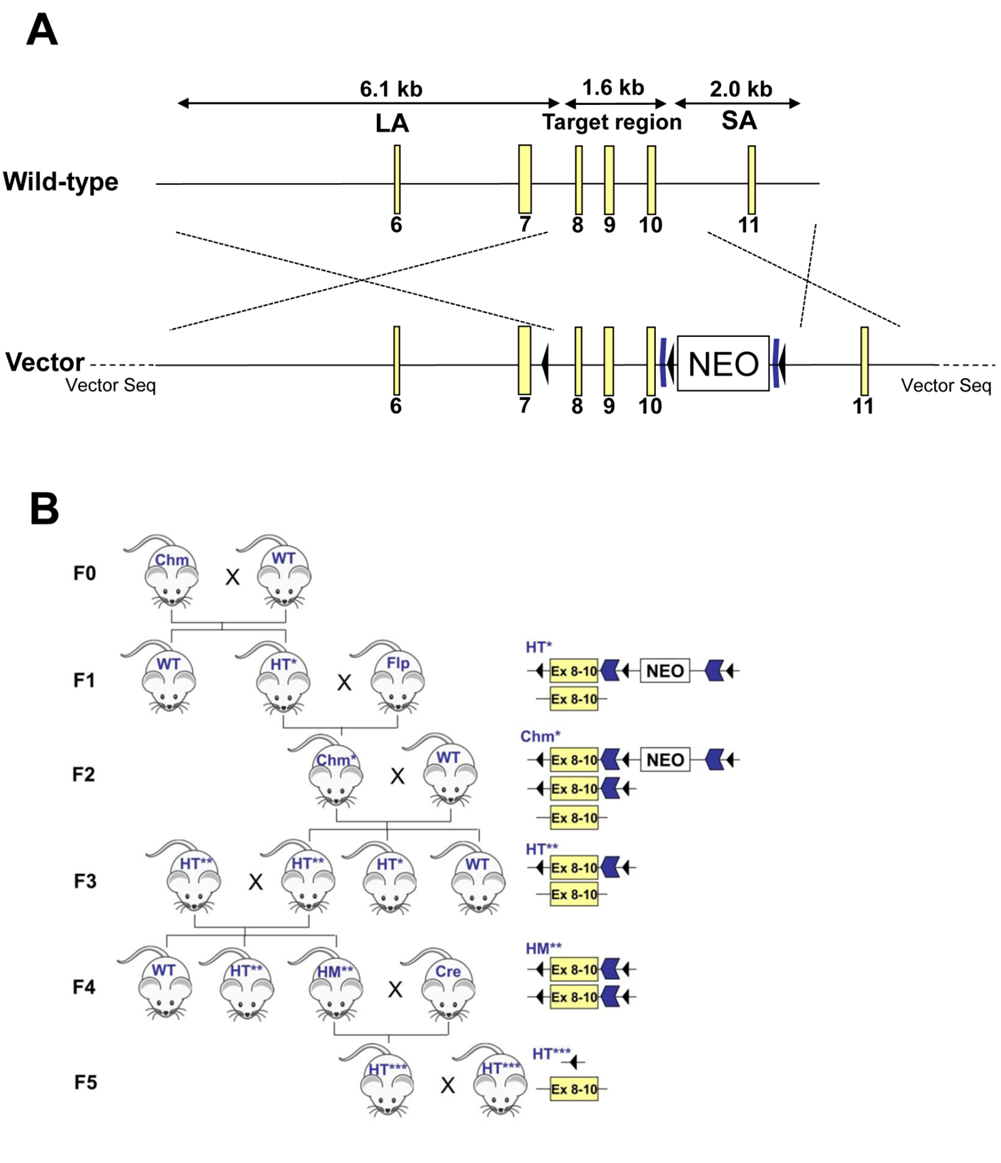
Supplemental Figures

**Figure S1. Targeting strategy and mating scheme for disruption of *Adamts3* in mouse**.

**A.** Structures of the *Adamts3* gene and the targeting vector are shown. The vector contains 6.1 kb and a 2.0 kb of *Adamts3* sequence in its long (LA) and short arm (SA), respectively. The target region (1.6 kb) is preceded by a *LoxP* sequence (black triangle) and followed by a *Neo* cassette flanked by *FRT* (blue vertical bars) and *LoxP* sequences on both sides. A 9.72 kb sequence extending from intron 5 to intron 11 of *Adamts3* was subcloned from a C57Bl/6 BAC clone. The *Adamts3* inactivation strategy comprised introduction of *LoxP* sites in intron 7 and intron 10 in order to allow the conditional removal of the sequence located between the two sites, which shifts the reading frame of the downstream mRNA sequence due to excision of exons 8, 9 and 10. The final targeting vector was engineered such that the short homology arm extends 2.0 kb to the 5’-end of the LoxP/FRT-flanked Neo cassette (in intron 10), while the long homology arm extends 6.1 kb to the 3’-end of the single *LoxP* site inserted (in intron 7). The targeting vector was electroporated into IC1 C57Bl/6 ES cells. The selected ES cells were then injected in blastocysts before insertion of the mixed-genotype blastocysts in foster mother. The entire procedure was performed by inGenious Targeting Laboratory, Inc, Stony Brook, NY 11790-3350, USA. **B.** Mating scheme for conditional disruption of *Adamts3*. F0 chimera mice were mated with wild-type mice to obtain F1 mice heterozygous for the floxed *Adamts3* allele. F1 heterozygous mice were first crossed with B6;SJL-Tg(ACTFLPe) 9205Dym/J mice (The Jackson Laboratory, USA, Stock No 003800) in order to delete FRT-flanked *Neo* cassette by action of Flp recombinase. Homozygous “Neo-deleted” mice (*Adamts3^lox/lox^*) were then crossed with B6.C-Tg(CMV-cre)1Cgn/J (The Jackson Laboratory, USA, Stock No 006054) allowing deletion of the *LoxP*-flanked region in all mouse tissues, including germ cells. F5 heterozygous mice were intercrossed to obtain homozygous mice. The expected genotype from each cross is indicated on the right. Chm: Chimera; WT: Wild-type (*Adamts3^+/+^*); HT: Heterozygous (*Adamts3^+/-^*); HM: Homozygous (*Adamts3^-/-^*); Flp: mice expressing Flp recombinase; Cre: mice with ubiquitous expression of Cre recombinase.


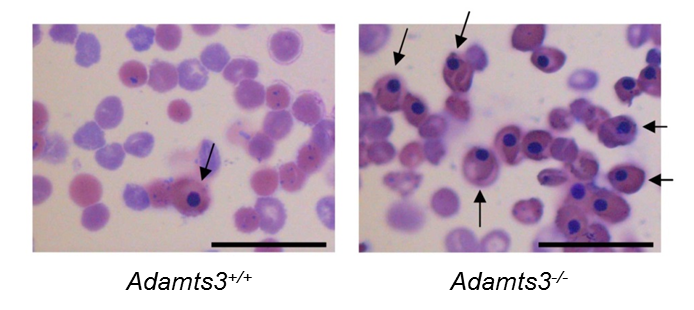


**Figure S2. Blood smears from E14.5 *Adamts3^+/+^* and *Adamts3^-/-^* embryos after May-Grunwald Giemsa staining.** Note the greater proportion of nucleated erythrocytes (arrows) in the *Adamts3^-/-^* smear (Scale bars =25µm).

**
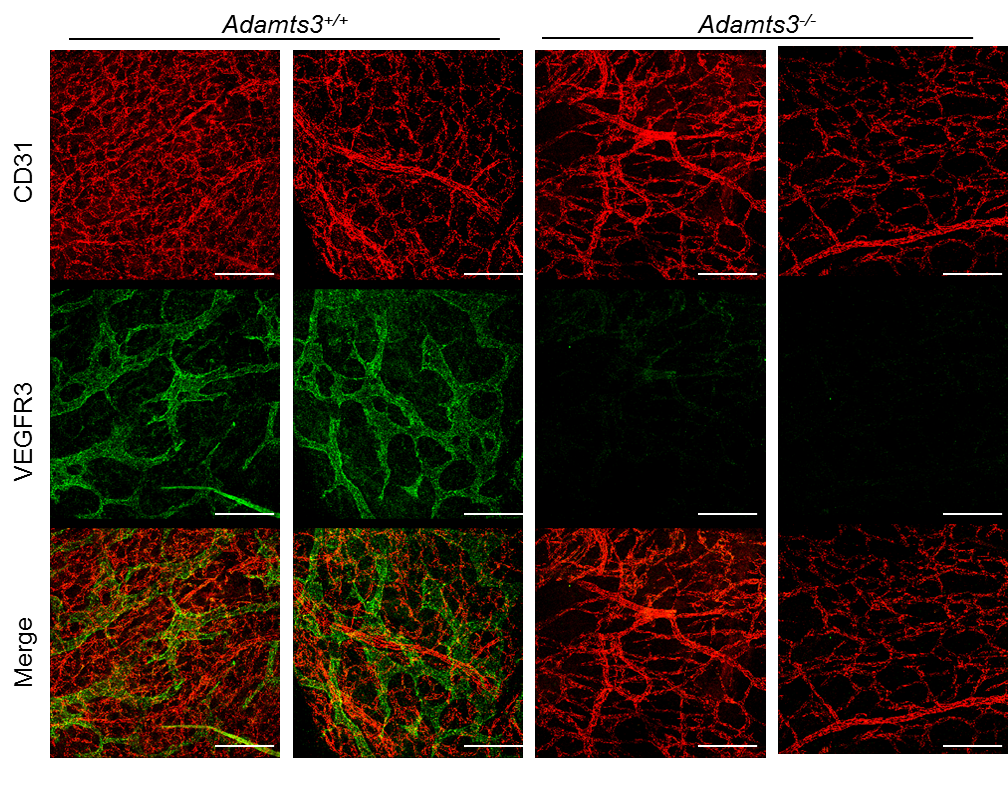
**

**Figure S3. Absence of lymphatics in the dorsal skin of *Adamts3^-/-^*mice.**

The dorsal skin of two different *Adamts3^+/+^* and *Adamts3^-/-^* E14.5 embryos was dissected, fixed and processed for the visualization of blood vessels (red, CD31-antibody) and lymphatics (green, VEGFR3-antibody). Merged pictures are provided in the bottom panels. No lymphatic vessels were observable in *Adamts3^-/-^* skin whereas the vascular network was unaffected. Scale bar = 200µm.


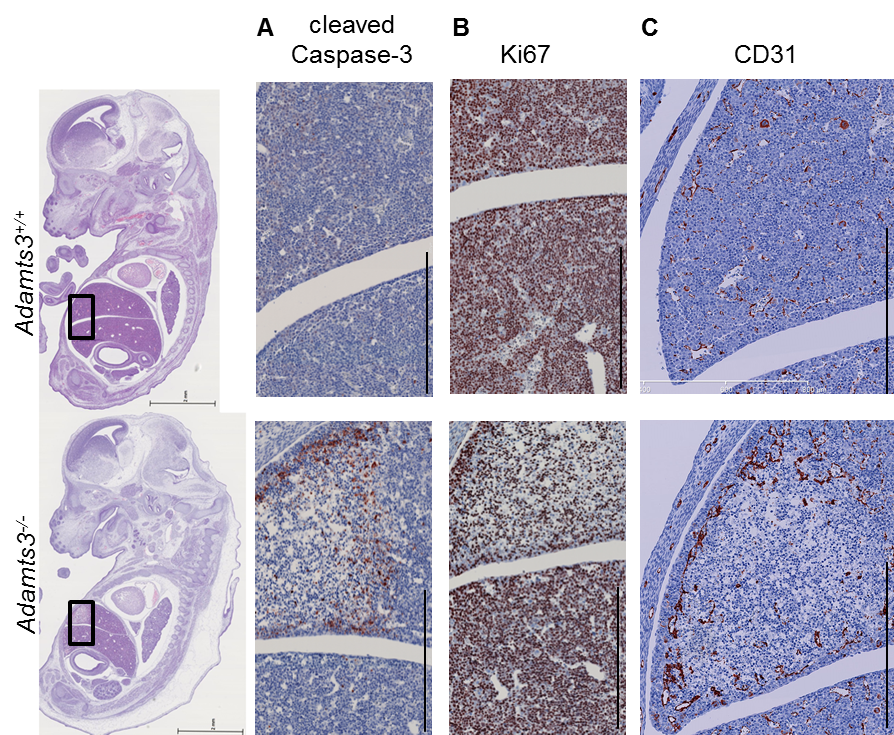


**Figure S4. Immunohistologic analysis of liver defects in E14.5 *Adamts3^-/-^* embryos.**

Sections showing affected areas in the liver were immunostained with antibodies specific for cleaved caspase-3 (**A**), Ki-67 (**B**) and CD31 (**C**), as markers of apoptotic cells, proliferating cells and endothelial cells, respectively. Enlargement of blood vessel is observed at the border of the apoptotic area. Scale bars = 400µm.


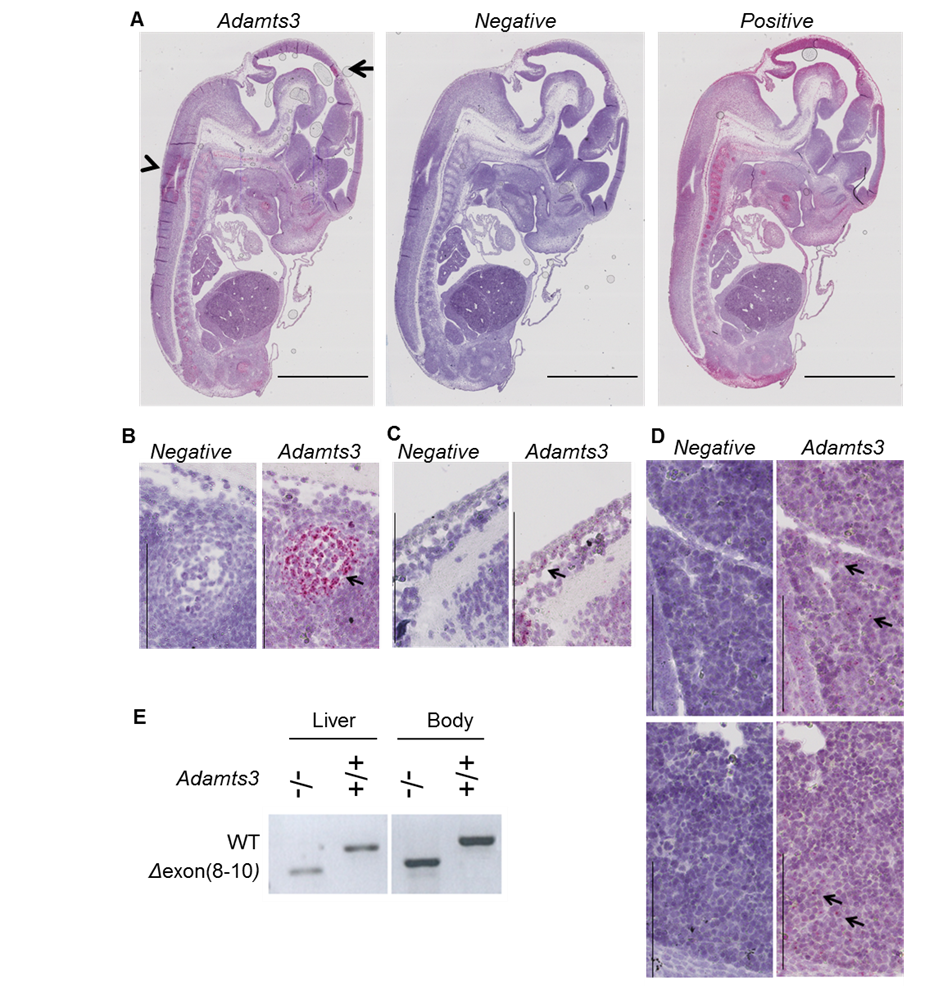


**Figure S5. Evaluation of *Adamts3* expression in the mouse embryo.**

**A**. Sections from E13.5 embryos were hybridized with *Adamts3* probes and with negative and positive control probes (see Methods). Positive signal is indicated by the presence of small red dots. At low magnification (Scale bars = 2mm) red staining is mainly seen in the central nervous system and the cartilage (arrow). **B-D.** Enlarged views (Scale bars = 200µm) of specific tissues. Cartilage (**B**) is strongly positive for *Adamts3*. Specific staining is also observed in the dermis (**C**) and in some liver cells (**D**). **E**. RT-PCR evaluation of *Adamts3* mRNA expression in liver and body (without head and viscera) of *Adamts3^+/+^* and *Adamts3^-/-^* embryos at E14.5. The forward and reverse oligonucleotides (specific to sequences in exon7 and 12, respectively) amplified full size *Adamts3* mRNA in *Adamts3^+/+^* tissues and mRNA lacking the sequence of exons 8-10 in *Adamts3^-/-^* tissues.

**
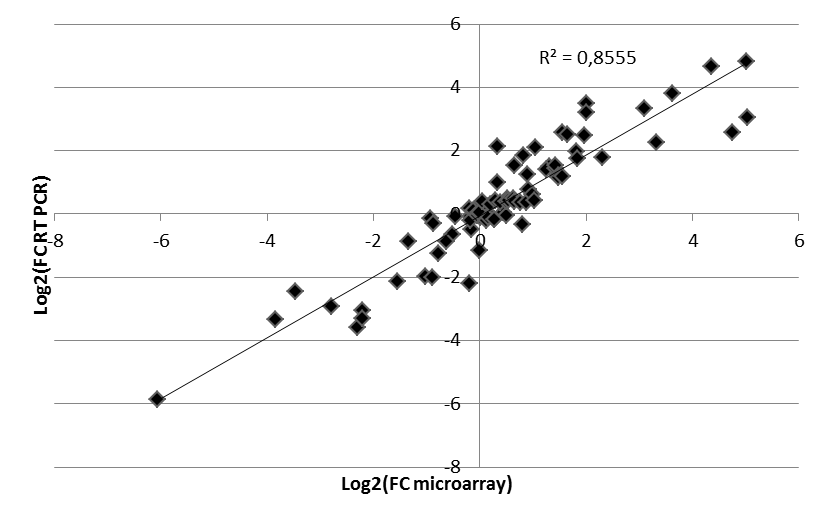
**

**Figure S6.** **Microarray data validation**.

mRNA from *Adamts3^+/+^* and *Adamts3^-/-^* livers (at E13.5, E14.0 and E14.5) and “bodies” (at E13.5 and E14.0) were RT-PCR amplified using 30 different pairs of primers (*Acta2, Adamts2, Apoa2, Atp2a1, Capn6, Ccl21, Col1a1, Col3a1, Col5a1, Col6a1, Cox6a2, Csrp3, Egr1, Eraf, Esm1, Gypa, H19, Hbby, Igfbp1, Igfbp5, Mpo, Mt1, Myh8, Myl1, Myl4, Mylpf, Stat3, Tnnc2, Trib3, Vegf-A*). Fold-changes (values for *Adamts3*^-/-^ / values for *Adamts3*^+/+^) were calculated and correlated to fold-changes determined from micro-arrays in log2 basis. When several efficient probes were available for a gene in the microarray, the mean value was reported on the graph. The R² value of the linear regression line was 0.86.


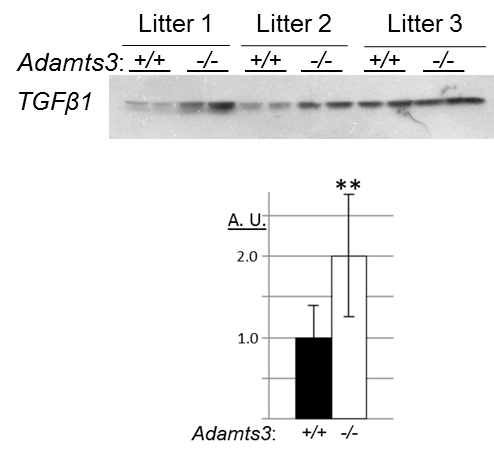


**Figure S7. TGFβ1 analysis in *Adamts3^-/-^* liver.**

Livers from six *Adamts3*^+/+^ and *Adamts3*^-/-^ embryos from three different litters (at E14.5) were collected and used to evaluate TGFβ1 levels by western blotting. Quantifications of the band intensities observed by ECL were normalized to the total protein content in the samples as determined by SYPRO-orange staining after reducing SDS-PAGE. The mean value for wild-type samples was arbitrarily set to 1. A one-way ANOVA was performed to evaluate the statistical significance of the results (p value = 0.01).


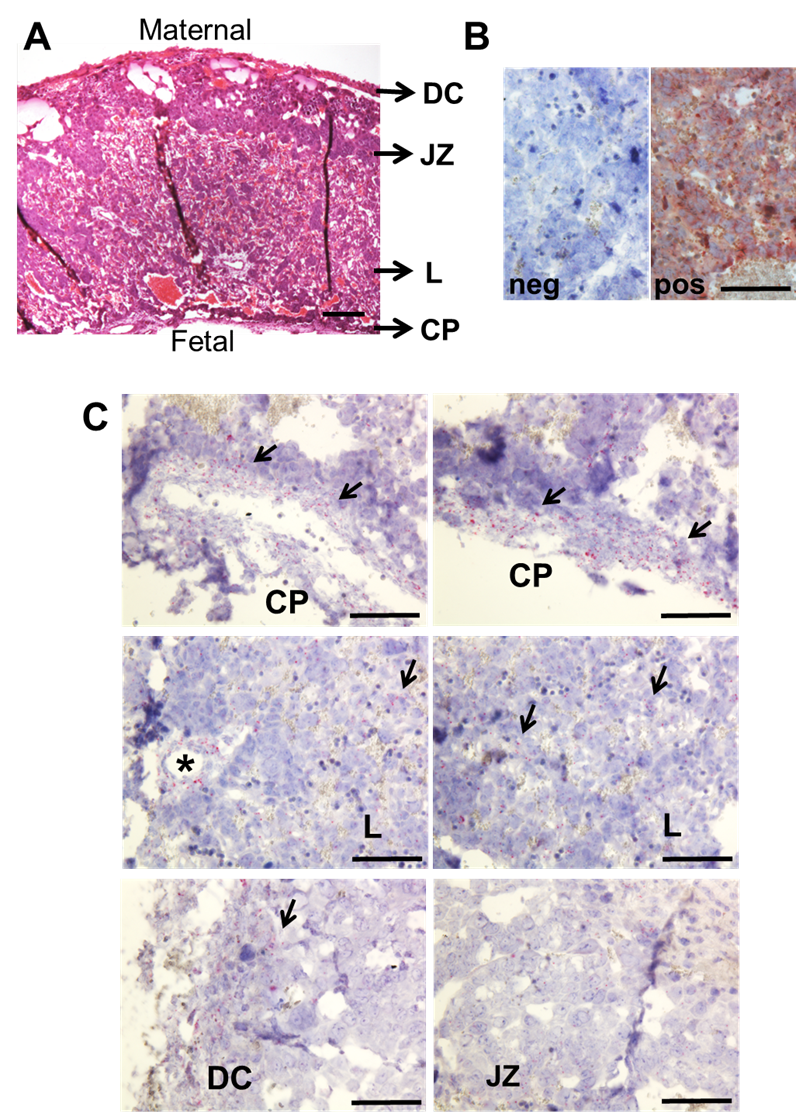
**Figure S8. Evaluation of *Adamts3* expression in mouse placenta.**

Sections of E12.5 placenta were stained with H&E (A) or were hybridized with negative and positive control probes (B) and with Adamts3 probes (C). Positivity is marked by the presence of small red dots (indicated by arrows). The strongest specific staining is observed in the chorionic plate (CP). Staining is also seen throughout labyrinth tissue (L), in particular in cells surrounding bigger blood vessels (see asterisk), and in the layer of decidual cells (DC). Cells of the junctional zone (JZ) show the weakest staining. Scale bars = 200μm (A) or 100 μm (B and C).


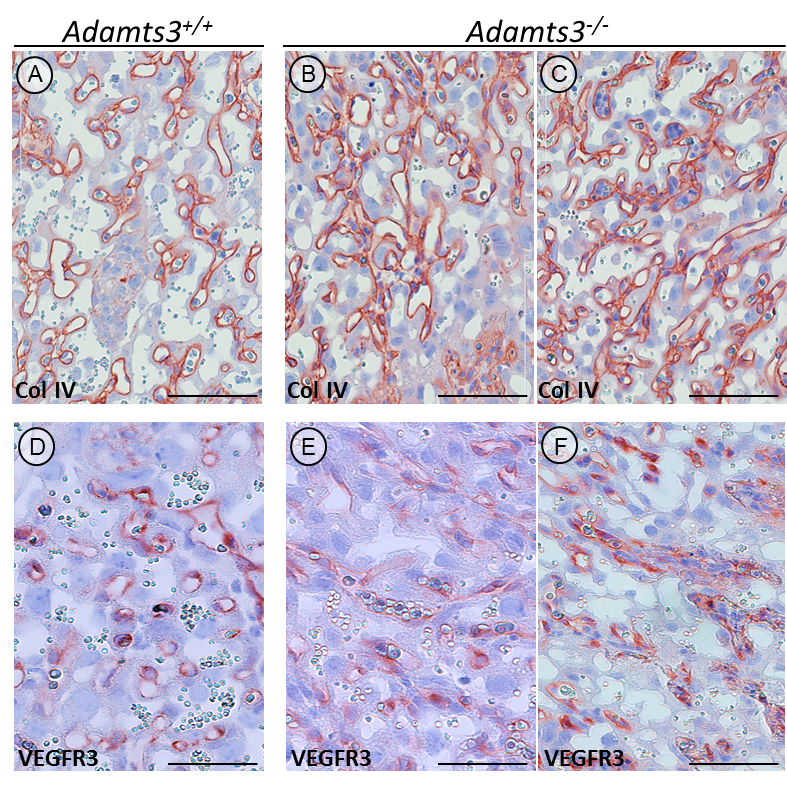


**Figure S9. Characterization of the blood vessels present in the labyrinthine layer of the placenta.** Transversal sections were performed in E14.5 placenta (*Adamts3^+/+^* in A and D; *Adamts3^-/-^* in B, C, E, F). All the pictures were taken in the labyrinthine layer where the blood vessels formed by endothelial cells and containing embryonic blood are present. Blood vessels are lined by a basement membrane (stained in brown) as determined by type IV collagen staining (A-C). Endothelial cells forming the vessels are positive for VEGF-R3 (D-F). Variable levels of VEGF-R3 expression in the endothelial cells are suggested by the variable staining intensities. Nuclei are counterstained in blue. Scale bars: 50 µm.

**
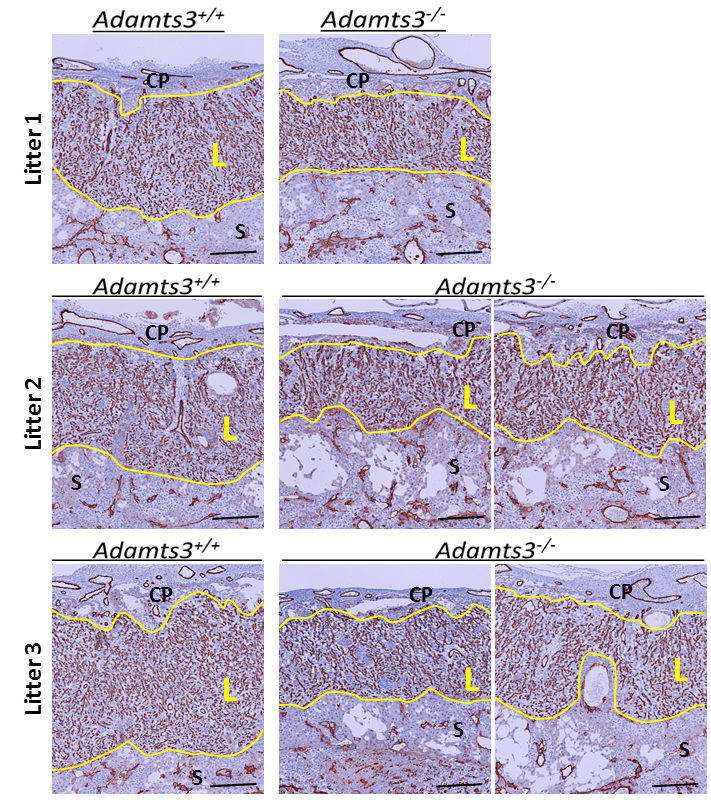
**

**Figure S10. Characterization of the labyrinthine layer in the placenta of Adamts3^+/+^ and Adamts3^-/-^ embryos.** Transverse sections, performed in the middle of 8 placentas from three different litters, were stained using an anti-CD31 antibody. Low magnification pictures were taken in the center of the section, as evidenced by the presence of the chorionic plate (CP). The labyrinthine layer (L, delineated by the yellow lines), which is clearly identified by its high blood vessel density, is significantly reduced in Adamts3^-/-^ placentas. Bars = 300 µm. CP: chorionic plate. S: spongiotrophoblast layer.
