# Supplementary material for: ADAMTS3 activity is mandatory for embryonic lymphangiogenesis and regulates placental angiogenesis
Source: Angiogenesis. 2015 Oct 7;19:53–65. doi: 10.1007/s10456-015-9488-z (PMC4700087; doi:10.1007/s10456-015-9488-z)
Supplement: Supplementary file 2 — Supplementary material 2 (DOCX 58 kb) [file 10456_2015_9488_MOESM2_ESM.docx]

## Supplemental tables

**Supplemental Table 1.** Microarray analysis on E13.5 livers from an *Adamts3*^-/-^ embryo compared to an *Adamts3*^+/+^ embryo from the same litter. Values for *Adamts3*^+/+^ (+/+) and *Adamts3*^-/-^ (-/-) embryos are given in A.U. Only genes with a Fold Change (FC, *Adamts3*^-/-^ / *Adamts3*^+/+^ratio) >2 or < 0.5 are reported.

| SYMBOL | +/+ | -/- | **FC** | DEFINITION |
| --- | --- | --- | --- | --- |
| Esm1 | 182 | 510 | **2.8** | endothelial cell-specific molecule 1 |
| Xlr4a | 299 | 789 | **2.6** | X-linked lymphocyte-regulated 4A |
| Gsta3 | 1545 | 3082 | **2.0** | glutathione S-transferase, alpha 3, transcript variant2. |
| Plp1 | 154 | 301 | **2.0** | proteolipid protein (myelin) 1 |
| Myl4 | 695 | 376 | **0.5** | myosin, light polypeptide 4 |
| Fech | 1839 | 990 | **0.5** | ferrochelatase |
| C030025P15Rik | 765 | 412 | **0.5** |  |
| Tpm2 | 852 | 458 | **0.5** | tropomyosin 2, beta |
| Actn2 | 328 | 175 | **0.5** |  |
| Ear2 | 438 | 233 | **0.5** | eosinophil-associated, ribonuclease A family, member 2 |
| Myh8 | 229 | 120 | **0.5** | myosin, heavy polypeptide 8, skeletal muscle, perinatal |
| Prg3 | 368 | 189 | **0.5** | proteoglycan 3 |
| Pnpo | 298 | 148 | **0.5** | pyridoxine 5'-phosphate oxidase |
| Myl1 | 2487 | 1213 | **0.5** | myosin, light polypeptide 1 |
| Ear12 | 1338 | 640 | **0.4** | eosinophil-associated, ribonuclease A family, member 12 |
| Actc1 | 295 | 130 | **0.4** | actin, alpha, cardiac muscle 1 |
| Ear10 | 923 | 365 | **0.4** | eosinophil-associated, ribonuclease A family, member 10 |
| Ear6 | 336 | 123 | **0.1** | eosinophil-associated, ribonuclease A family, member 6 |
| Ear4 | 1068 | 152 | **0.1** | eosinophil-associated, ribonuclease A family, member 4 |
| Prg2 | 1090 | 131 | **0.1** | proteoglycan 2, bone marrow |
| Ear2 | 3810 | 395 | **0.1** | eosinophil-associated, ribonuclease A family, member 2 |
| Ear2 | 3057 | 316 | **0.1** | eosinophil-associated, ribonuclease A family, member 2 |
| Ear2 | 1393 | 143 | **0.1** | eosinophil-associated, ribonuclease A family, member 2 |
| Ear3 | 3733 | 338 | **0.1** | eosinophil-associated, ribonuclease A family, member 3 |

**Supplemental Table 2.** Genes characterized by Fold Changes (FC, *Adamts3*^-/-^ / *Adamts3*^+/+^ratios) that progressively increase in E13.5, E14.0 and E14.5 livers. No gene was observed with a progressive decreased expression. Dark Red = FC increase over 2; Light red = FC increase between 1.5 and 2. Grey boxes identify genes involved in glucose homeostasis.

| **PROBES** | **FC: 13.5** | **FC: 14.0** | **FC: 14.5** |  |
| --- | --- | --- | --- | --- |
| Actb | 0,9 | 1,8 | 2,4 | actin, beta, cytoplasmic |
| Adamts2 | 1,0 | 1,6 | 2,4 | Adamts2 |
| Adh1 | 1,1 | 1,6 | 2,5 | alcohol dehydrogenase 1 (class I) |
| Adssl1 | 1,0 | 1,6 | 2,0 | adenylosuccinate synthetase like 1 |
| Aldoa | 1,2 | 2,6 | 4,8 | aldolase A, fructose-bisphosphate |
| Ampd2 | 0,9 | 1,9 | 2,5 | adenosine monophosphate deaminase 2 (isoform L) |
| Arhgdib | 1,0 | 1,7 | 2,0 | Rho, GDP dissociation inhibitor (GDI) beta |
| Asah3l | 1,5 | 2,8 | 2,0 | N-acylsphingosine amidohydrolase 3-like |
| Atp2a3 | 1,5 | 1,9 | 2,2 | ATPase, Ca++ transporting, ubiquitous |
| Bhlhb2 | 1,5 | 2,7 | 2,3 | basic helix-loop-helix domain containing, class B2, DEC1 |
| Capn6 | 1.0 | 1,6 | 3,6 | calpain 6 (Capn6) |
| Cebpb | 1.0 | 1,8 | 2,2 | CCAAT/enhancer binding protein (C/EBP), beta |
| Col1a1 | 0,8 | 1,8 | 3,0 | procollagen, type I, alpha 1 |
| Col5a1 | 1,1 | 1,7 | 2,4 | procollagen, type V, alpha 1 |
| Col6a1 | 1,3 | 2,0 | 5,4 | procollagen, type VI, alpha 1 |
| Col6a3 | 1,1 | 2,0 | 3,8 | procollagen, type VI, alpha 3 |
| Cpm | 1,0 | 1,6 | 2,3 | PREDICTED: carboxypeptidase M |
| Csnk1d | 1,2 | 1,6 | 2,4 | casein kinase 1, delta (Csnk1d) |
| Ctgf | 1,3 | 1,5 | 2,4 | Connective tissue growth factor, CCN2 |
| Ctsg | 0,9 | 1,6 | 2,9 | cathepsin G |
| Cxcl1 | 1,0 | 2,4 | 5,9 | chemokine (C-X-C motif) ligand 1 |
| Cxcl10 | 1,3 | 1,8 | 2,6 | chemokine (C-X-C motif) ligand 10 |
| Ddx3y | 1,6 | 1,4 | 3.0 | DEAD (Asp-Glu-Ala-Asp) box polypeptide 3, Y-linked |
| Dusp6 | 1,1 | 1,5 | 2,3 | dual specificity phosphatase 6 |
| Egln3 | 1,3 | 2.0 | 7,3 | EGL nine homolog 3/PHD3 |
| Egr1 | 1,4 | 2,0 | 2,0 | early growth response 1 |
| Eif4ebp1 | 1,3 | 1,8 | 3.0 | eukaryotic translation initiation factor 4E binding prot 1 |
| Eno | 0.9 | 1.4 | 2.3 | Enolase |
| Esm1 | 2,8 | 4,0 | 8,5 | endothelial cell-specific molecule 1 |
| Ets2 | 1,0 | 1,7 | 2,1 | E26 avian leukemia oncogene 2, 3' domain |
| F13a1 | 1,1 | 1,7 | 2,2 | coagulation factor XIII, A1 subunit |
| Fam132b | 0,9 | 2,8 | 1,8 | family with sequence similarity 132, member B, Myonectin |
| Flt1 | 1,4 | 1,7 | 2,1 | FMS-like tyrosine kinase 1 |
| GAPDH | 1.0 | 1.3 | 1.8 | glyceraldehyde-3-phosphate dehydrogenase |
| Gata6 | 0,9 | 2,0 | 1,5 | GATA binding protein 6 |
| Grhpr | 1,2 | 1,6 | 1.6 | Glyoxylate reductase |
| Gnas | 1,4 | 1,3 | 2,6 | guanine nucleotide binding protein, alpha stimulating |
| Golga2 | 1,1 | 1,4 | 2,0 | golgi autoantigen, golgin subfamily a, 2 |
| Gpi1 | 1,2 | 1,7 | 3,7 | glucose phosphate isomerase 1 |
| Gstm2 | 1.0 | 1,6 | 2,1 | glutathione S-transferase, mu 2 |
| Gtl2 | 1,2 | 1,5 | 2,4 | gene trap locus 2 |
| Hamp | 1,3 | 1,6 | 3,3 | hepcidin antimicrobial peptide |
| Hba-x | 0,6 | 1,3 | 3.0 | hemoglobin X, alpha-like embryonic chain |
| Hbb-y | 0.8 | 1.7 | 3.1 | hemoglobin Y, beta-like embryonic chain |
| Hist1h4m | 0,8 | 1,2 | 2,1 | histone cluster 1, H4m |
| Hmha1 | 1,3 | 2,4 | 1,9 | histocompatibility (minor) |
| Ifitm1 | 1.0 | 1,4 | 2,5 | interferon induced transmembrane protein 1 |
| Ifitm3 | 1,2 | 1,6 | 3,3 | interferon induced transmembrane protein 3 |
| Itga2b | 1,3 | 2,2 | 1,6 | integrin alpha 2b |
| Junb | 1,2 | 1,8 | 2,8 | Jun-B oncogene |
| Ldha | 1.1 | 1.9 | 1.5 | Lactate dehydrogenase |
| Lamc1 | 1,3 | 1,6 | 2,4 | laminin, gamma 1 |
| Lcn2 | 0,7 | 1,7 | 3,9 | lipocalin 2 |
| Lgals3 | 0,9 | 1,9 | 2,8 | lectin, galactose binding, soluble 3 |
| Mapkapk3 | 1,0 | 1,6 | 2,0 | mitogen-activated protein kinase-activated protein kinase 3 |
| Mpo | 1,1 | 1,6 | 1,8 | myeloperoxidase, nuclear gene encoding mitochondrial prot |
| Ms4a3 | 1,0 | 1,6 | 1,9 | membrane-spanning 4-domains, subfamily A, member 3 |
| Mt2 | 1,2 | 2,7 | 1,6 | metallothionein 2 |
| Myo1f | 1,0 | 1,6 | 2,1 | myosin IF |
| Nfkbiz | 1,2 | 1,6 | 2,2 | nfkappa light polyp gene enhancer in B-cells inhibitor, zeta |
| Nisch | 1,5 | 1,8 | 3,7 | Nischarin |
| Nupr1 | 1,0 | 1,5 | 3,3 | nuclear protein 1 |
| P4ha2 | 1,3 | 1,7 | 2,9 | proline 4-hydroxylase, alpha II polypeptide |
| Pecam | 1.0 | 1.4 | 1.7 | platelet/endothelial cell adhesion molecule 1, CD31 |
| PFKl | 1.1 | 1.6 | 1.6 | Phosphofructokinase liver specific |
| PFKp | 1.1 | 1.4 | 2.6 | Phosphofructokinase platelet specific |
| Pgam1 | 1.0 | 1,6 | 2,3 | phosphoglycerate mutase 1 |
| Pgk1 | 1,1 | 2,2 | 2,4 | phosphoglycerate kinase 1 |
| PKM2 | 1.1 | 0.9 | 4.3 | Pyruvate Kinase (muscle) |
| Pmp22 | 1,2 | 1,5 | 2,4 | peripheral myelin protein 22 |
| Prf1 | 1,5 | 1,2 | 4.0 | perforin 1 (pore forming protein) |
| Prtn3 | 1,1 | 2,0 | 2,9 | proteinase 3 |
| PDK1 | 1.0 | 2.1 | 1,3 | Pyruvate dehydrogenase kinase |
| Rhbdl2 | 1,8 | 1,7 | 3,5 | rhomboid, veinlet-like 2 |
| S100a9 | 0,9 | 2,3 | 2,0 | S100 calcium binding protein A9 (calgranulin B) |
| Scarf2 | 1,2 | 1,6 | 3,0 | scavenger receptor class F, member 2 |
| Siat7c | 1,5 | 2,8 | 3,2 | alpha-2,6-sialyltransferase ST6GalNAc III |
| Slc2a3 | 1,5 | 1,6 | 3,0 | solute carrier family 2/Glut3 |
| Slc7a3 | 1,4 | 1,7 | 2,2 | solute carrier family 7, member 3 |
| St3gal1 | 1,1 | 1,6 | 2,2 | ST3 beta-galactoside alpha-2,3-sialyltransferase 1 |
| Stat3 | 1,1 | 1,6 | 2,4 | signal transducer and activator of transcription 3 |
| Trem3 | 0,8 | 1,6 | 2,3 | triggering receptor expressed on myeloid cells 3 |
| Trib3 | 1,9 | 2,9 | 6,4 | tribbles homolog 3 |
| Tpi | 1.2 | 1.6 | 1.8 | Triosephosphate isomerase |
| Tssc8 | 1,6 | 1,8 | 2,2 | tumor-suppressing subchromosomal transferable fragment 8 |
| Vav1 | 1,1 | 1,9 | 2,2 | vav 1 oncogene |
| Vegfa | 1,4 | 2,0 | 2,6 | vascular endothelial growth factor A |
| Ybx3 | 1.0 | 1,7 | 2,4 | Y box protein 3 |
| Zfp36 | 1,1 | 1,6 | 2,1 | zinc finger protein 36 |

**Supplemental Table 3.** Determination of the mean diameter (in µm) of the CD31-positive vessels containing the embryonic blood in the labyrinthine layer of the placenta. Cross sections were performed in the middle of the placenta and stained with an anti-CD31 antibody. All the blood vessels with a lumen and “circularity” (longest diameter/shortest diameter) between 0.5 and 1 were used for the quantification (NanoZoomer Digital Pathology software, Hamamatsu). Only the shortest length was considered for the determination of blood vessel diameter in order to prevent any overestimation caused by sections that do not cross vessels perpendicularly.

|  | Genotype | Mean diameter  (in µ +/- SD) | Measured vessels (n) | Vessel diameter (%)  (*Adamts3^-/-^ /Adamts3^+/+^*) |
| --- | --- | --- | --- | --- |
| Litter 1 | *Adamts3^+/+^* | 10.7 +/- 2.7 | 224 |  |
|  | *Adamts3^-/-^* | 9.5 +/- 2.1 | 225 | 88.8 % |
| Litter 2 | *Adamts3^+/+^* | 9.6 +/- 2.1 | 215 |  |
|  | *Adamts3^-/-^* | 7.9 +/- 2.1 | 288 | 82.3 % |
| Litter 3 | *Adamts3^+/+^* | 9.5 +/- 2.2 | 328 |  |
|  | *Adamts3^-/-^* | 8.5 +/- 2.5 | 150 | 89.5 % |
| Litter 4 | *Adamts3^+/+^* | 10.3 +/- 2.6 | 311 |  |
|  | *Adamts3^-/-^* | 8.4 +/- 2.0 | 136 | 81.6 % |
| **Total** | ***Adamts3^+/+^*** | **10.0 +/- 0.6** | **1078** |  |
|  | ***Adamts3^-/-^*** | **8.6 +/- 0.7** | **799** | **86.0 % (p< 0.01)** |

**Supplemental Table 4.** Oligonucleotides used for genotyping. Fwd = forward primer; Rev = reverse primer.

|  | **target** | **sequence** |
| --- | --- | --- |
| P1 | *Adamts3*-exon10 Fwd | 5’ TGGCGTTGGCTACAAGATGTGTAC 3’ |
| P2 | *Adamts3*-intron10 Rev | 5’ GGCTATGTACCCAAAGTGTCTACT 3’ |
| P3 | *Adamts3*-intron7 Fwd | 5’ GCCAACAGTCTTTCTTCCAAAGTCA 3’ |
| P4 | *Neomycin* Fwd | 5’ CTGCTAAAGCGCATGCTCCAGAC 3’ |
| P5 | *Flp* Rev | 5’ CACTGATATTGTAAGTAGTTTGC 3’ |
| P6 | *Flp* Fwd | 5’ CTAGTGCGAAGTAGTGATCAGG 3’ |
| P7 | *cre* Fwd | 5’ ATCCGAAAAGAAAACGTTGA 3’ |
| P8 | *cre* Rev | 5’ ATCCCAGGTTACGGATATAGT 3’ |
